# Spatial competition dynamics between reef corals under ocean acidification

**DOI:** 10.1038/srep40288

**Published:** 2017-01-09

**Authors:** Rael Horwitz, Mia O. Hoogenboom, Maoz Fine

**Affiliations:** 1 The Mina and Everard Goodman Faculty of Life Sciences, Bar-Ilan University, Ramat-Gan 52900, Israel; 2 The Interuniversity Institute for Marine Sciences, Eilat 88103, Israel; 3 College of Science and Engineering and ARC Centre of Excellence for Coral Reef Studies, James Cook University, Townsville, Queensland 4811, Australia

## Abstract

Climate change, including ocean acidification (OA), represents a major threat to coral-reef ecosystems. Although previous experiments have shown that OA can negatively affect the fitness of reef corals, these have not included the long-term effects of competition for space on coral growth rates. Our multispecies year-long study subjected reef-building corals from the Gulf of Aqaba (Red Sea) to competitive interactions under present-day ocean pH (pH 8.1) and predicted end-of-century ocean pH (pH 7.6). Results showed coral growth is significantly impeded by OA under intraspecific competition for five out of six study species. Reduced growth from OA, however, is negligible when growth is already suppressed in the presence of interspecific competition. Using a spatial competition model, our analysis indicates shifts in the competitive hierarchy and a decrease in overall coral cover under lowered pH. Collectively, our case study demonstrates how modified competitive performance under increasing OA will in all likelihood change the composition, structure and functionality of reef coral communities.

Rising atmospheric carbon dioxide (CO_2_) has recently exceeded 400 ppm, the highest level in recorded history^1^. The resulting elevated sea surface temperature (SST) is accompanied by increased partial pressure of CO_2_ (*p*CO_2_) in the ocean, which changes the relative amounts of inorganic carbon (i.e., carbonate versus bicarbonate and dissolved CO_2_), ultimately making the ocean more acidic^2^. This process is known as ocean acidification (OA), and it is expected to have detrimental consequences on many marine ecosystems, including seagrass beds, kelp forests, tidal wetlands and mangroves^3^,^4^. It is important to note, however, that compared to open-ocean environments, pH changes in coastal waters derive from a complex interaction between anthropogenic CO_2_ emissions and dynamic regional to local drivers (e.g., watershed processes, nutrient inputs, changes in ecosystem structure and metabolism), all of which contribute towards human-driven impacts on seawater pH^5^.

Coral reefs are particularly at risk from OA because the skeletal growth (calcification) of corals fundamentally depends upon the availability of carbonate ions in seawater^6^. Corals play a critical role in reef construction, and provide essential structural complexity for thousands of fish and invertebrate species (e.g., ref. 7). An increasing body of evidence has revealed negative effects of OA on coral growth, reproduction and survivorship (e.g., refs 8 and 9). However, uncertainty over the consequences of OA at the community and ecosystem levels remains because, to date, the majority of studies of the effects of OA have excluded the potentially important effects of biological interactions that form the foundation of coral reef community dynamics, such as competition^10^. OA can mediate competition among species because it influences both the supply of resources and the demand for them. These coupled responses create a complex interplay among the physiological susceptibility of organisms to OA, the availability of resources, and the intensity of competition.

Competition is an important determinant of community structure in high-diversity ecosystems like rainforests and coral reefs^11^. The ability of corals to exploit and maintain a multi-dimensional space, both on the reef substratum (via planar growth) and water column (via vertical extension), depends on species morphology, growth rate, aggression ability and the surrounding environmental conditions^12^. Competitive relationships between coral taxa can be classified as “direct”, comprising of digestive activity and physical overgrowth, or “indirect”, through overtopping and allelopathy^12^. The competitive mechanism used in any interaction stems from specific life history traits and the surrounding environmental factors^13^. Traditionally, branching species, such as Acroporids and Pocilloporids, use physical overgrowth and overtopping, whereas massive forms employ digestive competition^12^. Fast- and slow-growing coral species can coexist because the speed at which branching corals grow is balanced by the aggressive nature of massive corals^14^.

Over the past few decades, numerous studies have assessed the outcome of competitive interactions between coral species on reefs (e.g., refs 12, 15 and 16), and have revealed that the competitive hierarchy of species, usually based on aggression ability ranking, can differ between locations^16^. Changes in environmental conditions can alter the outcome of competitive interactions between species (e.g., ref. 17) and there is growing evidence of altered species interactions under climate change in marine (e.g., refs 18 and 19) and terrestrial (e.g., ref. 20) environments. In one recent study, temperature stress lowered the competitive advantage of some corals to the point where many coral genera changed in abundance^21^. Likewise, OA studies have reported that acidified conditions differentially alter species’ competitive ability, such as in fish^22^ and crustose coralline algae^23^, to the extent that patterns of dominance shift. Another study showed how coral mortality increased two- to threefold under increased seawater *p*CO_2_ when competing with a common coral reef seaweed^24^. In contrast, elevated *p*CO_2_ did not alter effects of competition on the growth of hard corals competing with soft corals in a short-term experiment^25^. Understanding these impacts is crucial because changing competition dynamics can alter species relative abundances within communities^10^. Consequently, ecosystem functioning can be affected because different species make different contributions to reef structural complexity, primary production and accretion^26^, and to nutrient exchange between reefs and the open ocean^27^.

Given that OA can change energy allocation amongst major life functions (i.e., growth, fecundity, regenerative capability) and overall coral fitness^9^, we hypothesized that increasing acidity would alter the outcome of spatial competition within and between coral species. Global declines in coral cover, due to mass bleaching events and other drivers (e.g., ref. 28), would, however, be expected to compound the effects of competition encounter frequency between and among reef coral taxa, and lead to fewer and smaller reef areas of high-density coral cover. Nonetheless, this study accounts for many reef ecosystems throughout the world, which currently have high coral cover and healthy status, thus harboring frequent inter-coral competition. Competitive interactions also occur on reefs with low coral cover. This can, and does, occur because coral larvae prefer certain conditions and often settle very close to each other and compete for reef substrate^29^. Additionally, space suitable for colonization by hard corals can be very low on degraded reefs (e.g., due to macroalgal dominance) despite their low coral cover^30^. To date, only two short-term studies have investigated the effects of OA on competitive interactions, finding that competition had little to no effect on coral growth under acidified conditions over ~3–4 weeks^31^,^32^. However, long-term experiments are required to quantify competition outcomes because many corals grow slowly (particularly massive coral species known for their aggressive nature^12^), coral growth rates vary on seasonal cycles (e.g., ref. 33), and reversals in competition outcomes can occur over time^34^.

Here we describe the outcome of direct competitive interactions within (intraspecific competition) and between (interspecific competition) reef-building coral species maintained under acidified compared with ambient conditions for one year. Six common Indo-Pacific reef-building coral species (*Galaxea fascicularis, Pocillopora damicornis, Cyphastrea chalcidicum, Acropora variabilis, Porites lutea* and *Stylophora pistillata*) were chosen based on differences in their aggression ranking^16^, and to represent the different morphological groups that are commonly found on reefs (see Table 1). Species are referred to by genus names hereafter. Pair-wise interactions between neighboring corals were recorded over 345 days and compared between ambient (pH 8.1) and reduced pH [pH 7.6, ΔpH~0.5 from present-day, consistent with the IPCC RCP 8.5 scenario for 2100^35^] conditions (see Table S1 for experimental seawater parameters). In order to establish the term “competitive ability” and provide an ecologically relevant measure of realized growth and space capture on the coral reef, we used a common metric of surface area growth when comparing corals with different growth forms, i.e., rate of change in overall colony surface dimensions. This is an important determinant of colony fate and fitness, affecting fecundity^36^,^37^ and probability of mortality^38^. In order to scale-up the results of the experimental study to a natural field setting, we used the data from the competition experiment to parameterize a mathematical model describing spatial competition^39^. This approach enabled us to evaluate whether coral cover, species coexistence and hierarchies of competitive ability were altered under OA compared with present-day conditions.

## Results

### Experimental findings

**Figure 1:**
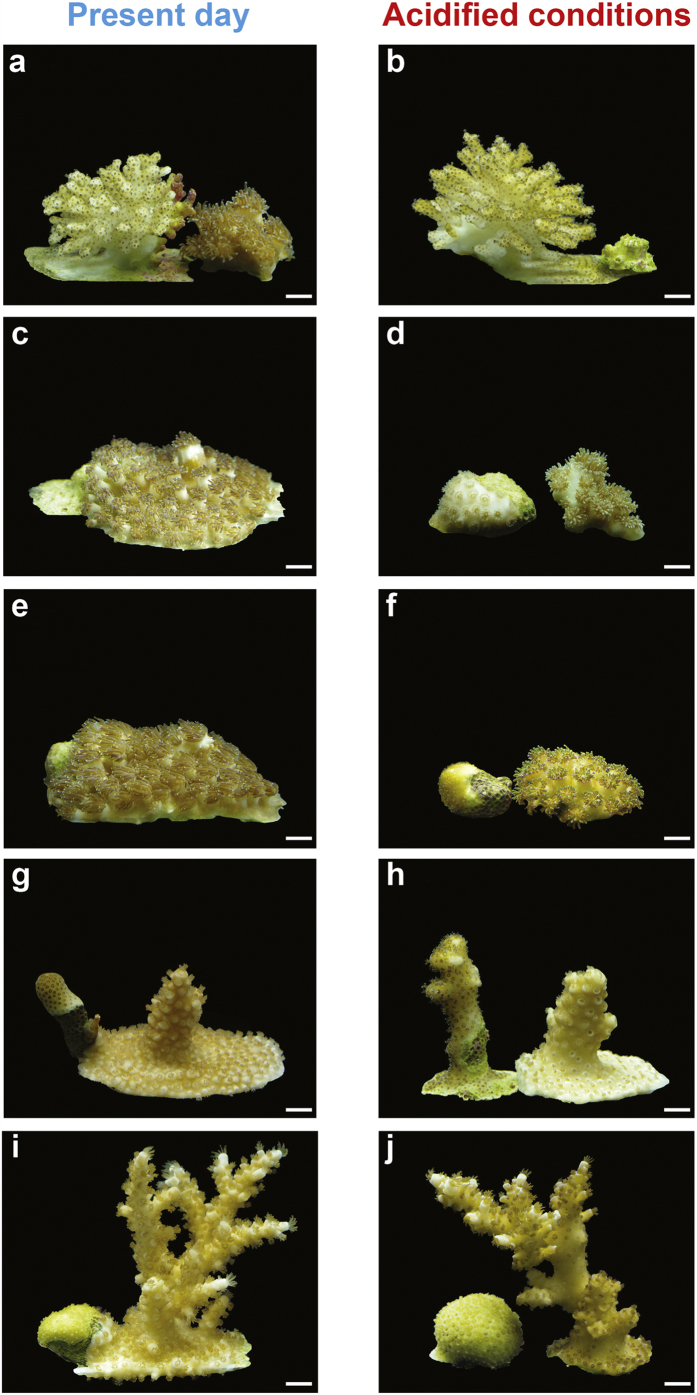
Corals growing under interspecific competition. Images show representative coral fragments from pair-wise interactions under present-day (pH 8.1) and acidified (pH 7.6) conditions for: (**a** and **b**) *Pocillopora damicornis* vs. *Galaxea fascicularis*. (**c** and **d**) *Cyphastrea chalcidicum* vs. *G. fascicularis*. (**e** and **f**) *Porites lutea* vs. *G. fascicularis*. (**g** and **h**) *Stylophora pistillata* vs*. Acropora variabilis*. (**i** and **j**) *P. lutea* vs. *A. variabilis*. Scale bar length is 1 cm.

**Figure 2:**
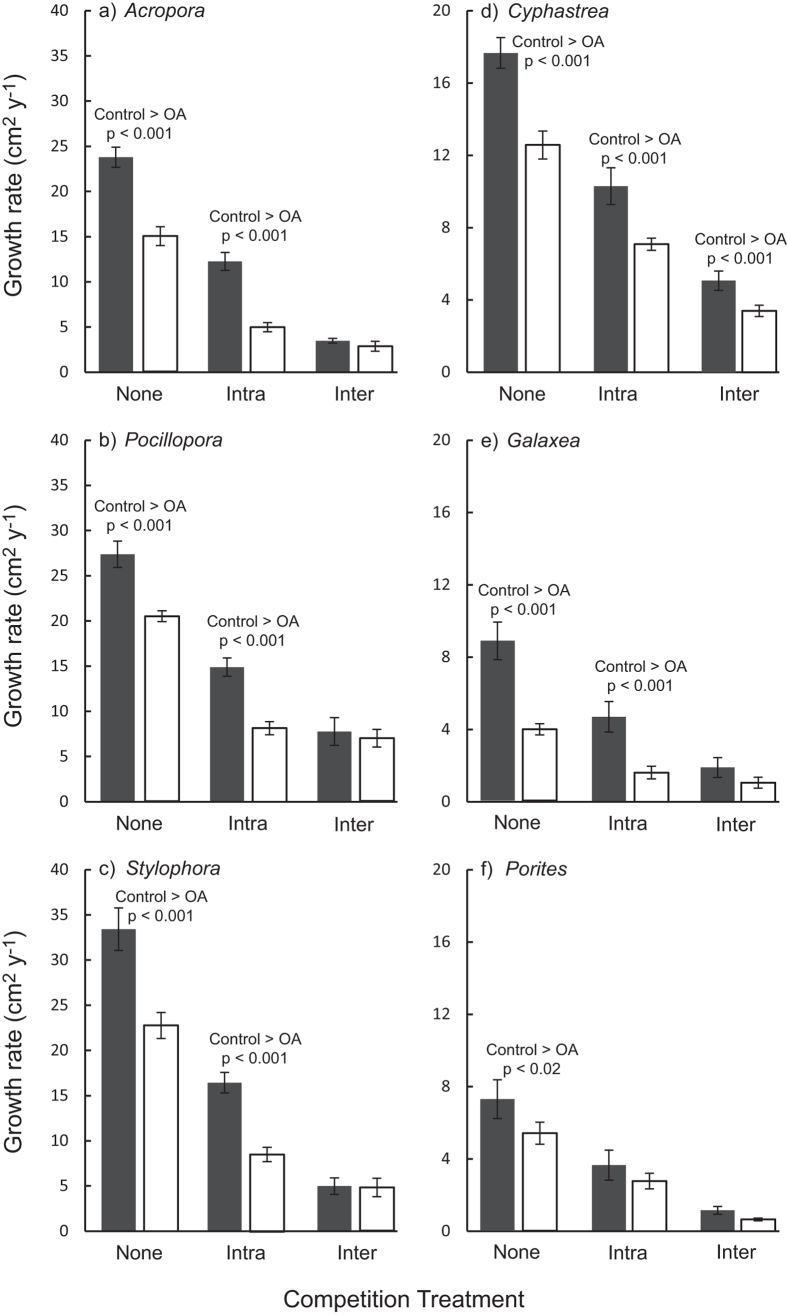
Coral growth under different types of competition. Corals grown in isolation (None) compared with corals’ growth with intra- and interspecific (inter-) competitors. (**a**) *Acropora variabilis,* (**b**) *Pocillopora damicornis*, (**c**) *Stylophora pistillata*, (**d**) *Cyphastrea chalcidicum,* (**e**) *Galaxea fascicularis*, and (**f**) *Porites lutea*. Shaded bars show growth under present-day (pH 8.1; ‘Ambient’; *n* = 6 for each bar) compared with acidified conditions (pH 7.6, open bars; ‘OA’; *n* = 6 for each bar) and error bars show standard deviation.

All of the coral colonies in our year-long experiment survived under both ambient and acidified treatments, and most increased in size, indicating that conditions remained within the physiological tolerance range of each species. For corals not involved in competition, growth rates were between 25% (for *Pocillopora* and *Porites*) and 55% (for *Galaxea*) lower under OA conditions compared with present-day conditions, reflecting an ecologically significant suppression of growth (Figs 1 and 2). The effect of competition on growth depended upon pH treatment but these effects manifested differently for different species (Table 2; ANOVA ‘Type of competition’, Treatment:Species:Competition interaction, F_10,180_ = 8.3, p < 0.001). Intraspecific competition had a lesser effect on coral growth rates compared with interspecific competition for all six study species under ambient conditions (Fig. 2, Tukey’s posthoc test, None > Intra > Inter for all species under ambient conditions, see Table S2). In contrast, for *Pocillopora* and *Galaxea*, growth suppression was the same under intra- and interspecific competition when grown under OA conditions (Fig. 2, Tukey’s posthoc test, None > Intra = Inter for *Pocillopora* and *Galaxea*, but None > Intra > Inter for the other species under reduced pH conditions, Table S2). Further comparisons of OA effects for different species under different types of competition revealed that growth was higher under ambient compared with OA conditions in all competition treatments for *Cyphastrea*; only in the absence of competition and under intraspecific competition for *Galaxea, Stylophora, Pocillopora* and *Acropora*; and only in the absence of competition for *Porites* (Fig. 2, Table S2). Collectively, these results indicate that the depressed growth rate observed during interspecific competition was too low to be depressed further by OA conditions.

We found no evidence that the magnitude of the effect of a particular heterospecific competitor on a particular species differed under OA compared with ambient conditions (Table 2; ANOVA ‘Identity of competitor’, Treatment:Species:Competitor interaction, F_19,300_ = 1.1, p = 0.39). Instead, the effect of pH treatment differed among species (regardless of competitor identity, Table 2, Treatment:Species interaction, F_5,300_ = 3.9, p < 0.01) with growth being higher under ambient compared with OA conditions for *Porites, Cyphastrea* and *Galaxea* involved in interspecific competition, but not for the other three species (Tukey’s posthoc test, Table S3). For example, *Galaxea* exhibited strikingly reduced growth under interspecific competition at lowered pH (Fig. 1a–f). We note that this result is generally consistent with our analysis for ‘Type of competition’ (Table 2), except for *Galaxea* for which the increased sample size in the ‘Identity of competitor’, analysis meant that the difference between pH treatments was significant (p < 0.001 in ‘Identity of competitor’, Table S3, compared with p = 0.05 in ‘Type of competitor’, Table S2).

**Figure 3:**
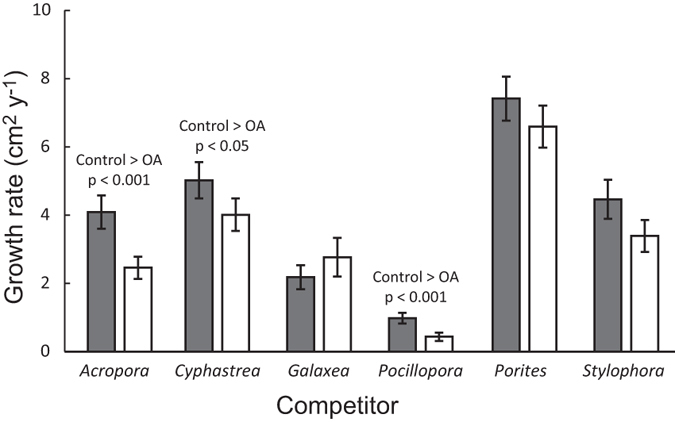
Coral growth in the presence of different competitors. Growth rate under present-day (pH 8.1, filled bars; ‘Ambient’) conditions is compared with acidified conditions (pH 7.6, open bars; ‘OA’). Data show mean growth of all species interacting with each competitor (*n* = 36 for each bar) and error bars show standard error.

**Figure 4:**
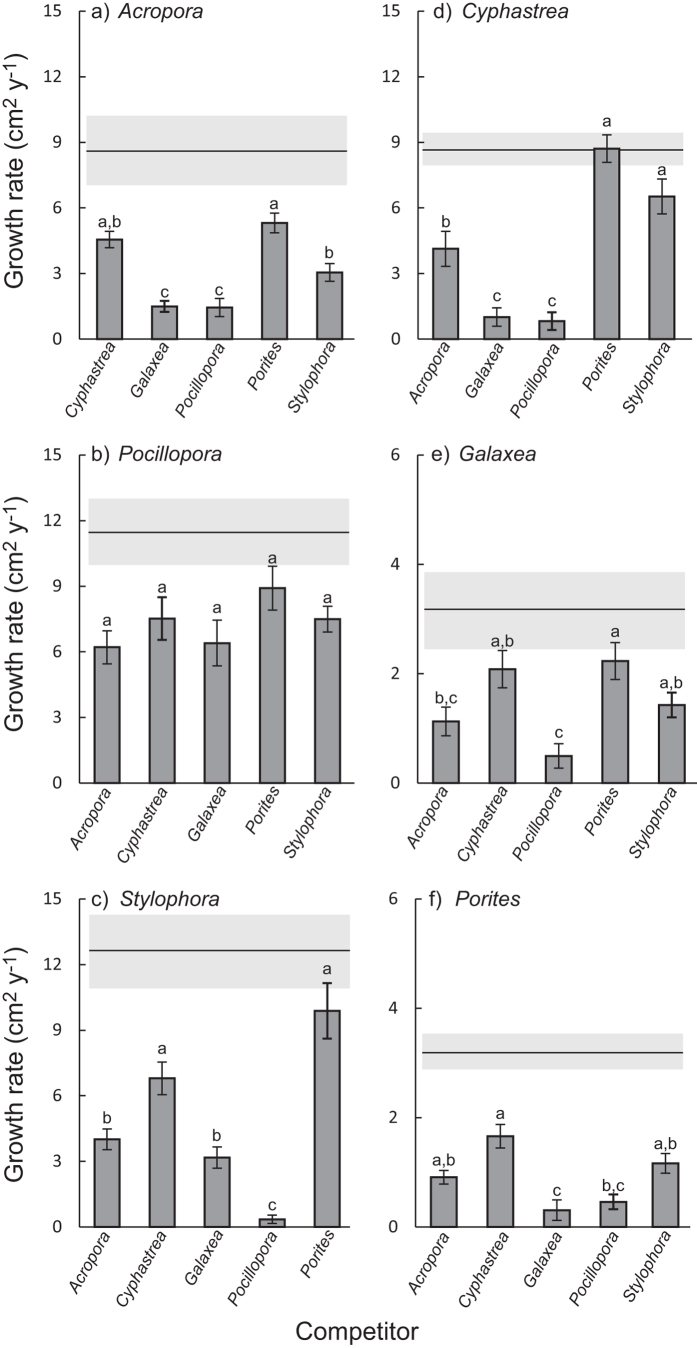
Coral growth under interspecific competition in the presence of different competitors. (**a**) *Acropora variabilis*, (**b**) *Pocillopora damicornis*, (**c**) *Stylophora pistillata*, (**d**) *Cyphastrea chalcidicum*, (**e**) *Galaxea fascicularis*, and (**f** ) *Porites lutea*. Data are pooled across pH treatments (*n* = 12 for each bar). In each panel, the solid horizontal line and shaded bar indicate mean and standard error of that species growing under intraspecific competition. Error bars show standard error and letters above bars indicate homogeneous subsets (for each species) based on post-hoc comparisons.

Our results also show that certain competitors suppressed the growth of all species they interacted with more severely under OA compared with ambient conditions (Table 2; ANOVA ‘Identity of competitor’, Treatment:Competitor interaction, F_5,300_ = 4.0, p < 0.01). The growth of all species when competing with *Acropora, Cyphastrea* and *Pocillopora* was lower, on average, under OA compared with present-day conditions (Fig. 3, Tukey’s posthoc test, p < 0.05), but reduced pH did not affect the outcomes of competitive interactions with *Galaxea* *Porites* or *Stylophora* (Fig. 3, Tukey’s posthoc test, p > 0.1). Regardless of pH treatment, different heterospecific competitors had varying effects on the growth of particular species (Table 2; ANOVA ‘Identity of competitor’, Species:Competitor interaction, F_19,300_ = 15, p < 0.001). Among the six study species, *Pocillopora* was the only one for which all five heterospecific competitors had the same effect on growth (Fig. 4, Table S3). In contrast, growth of *Acropora* was most strongly suppressed by *Galaxea* and *Pocillopora* (Fig. 4a), and a similar result was observed for *Cyphastrea* (Fig. 4d) and *Porites* (Fig. 4f).

### Modeling analysis

To assess whether the observed variation in strength of intra- and interspecific competition under OA conditions influenced the population dynamics of the study species, we parameterized and analyzed two-species competition models^39^ (see Materials and Methods). In the model, growth onto free space occurs by extension of overall colony dimensions and/or arrival of new individuals into the population, and is represented by a constant per-capita expansion rate, *b*_*i*_ for species *i*. Mortality occurs at a constant per capita rate, *d*_*i*_ for species *i*, which releases space that is then re-occupied through a lottery process (e.g., ref. 40). In this study, we assumed that background mortality was the same for all species but we note that this could be modified to incorporate additional complexity (e.g., by allowing mortality to vary between species based on bleaching susceptibility). The competitive ability of each species depends partly on its own intrinsic expansion rate (i.e., on *b*_*i*_), and also on the extent to which this expansion slows along the boundary between the two competing species. In the model, this process is expressed for species *i* as *c*_*ij*_*b*_*i*_where c_*ij*_ is a dimensionless coefficient expressing the proportional change in growth of species *i* when interacting with species *j.*

For each pair of species, we assessed whether species coexistence was possible under both ambient and lowered pH conditions [see Supplementary Information (SI1) for Matlab code and parameter estimates). In the model, stable coexistence between competitors occurs when the net rate of expansion of the inferior competitor is greater than the value of (*b*_*j*_*d*_*i*_ − *b*_*i*_*d*_*j*_)/(*c*_*ij*_*b*_*i*_ − *c*_*ji*_*b*_*j*_), and this value is, in turn, greater than the net expansion rate of the superior competitor^39^. Overall, results from the modeling showed that the fraction of space occupied by dominant species was smaller under OA in all cases, reflecting the general decrease in colony growth rates under lowered pH. In our results, conditions for species coexistence were only met under ambient conditions, and only for *Acropora* in competition with *Galaxea* (*f*_*Acropora*_ = 0.47, *f*_*Galaxea*_ = 0.44) and for *Acropora* in competition with *Pocillopora* (*f*_*Pocillopora*_ = 0.8, *f*_*Acropora*_ = 0.08). Using these results, we assessed the competitive hierarchy of the species by scoring the outcome of each pair-wise interaction with 2 points when a species totally outcompeted a heterospecific, 1 point when a species was more abundant than a heterospecific but both species persisted in the environment, 0 points for a subordinate where both species were persistent, and −2 points for subordinates that were excluded from the environment. Results showed a shift in the competitive hierarchy between ambient and lowered pH conditions, with *Porites* < *Cyphastrea* < *Stylophora* < *Galaxea* < *Acropora* < *Pocillopora* under ambient conditions and *Porites* < *Cyphastrea* < *Galaxea* < *Stylophora* < *Pocillopora* < *Acropora* under lowered pH (Table 3).

For comparison with the competitive hierarchy from the spatial competition model we ranked species based on their capacity to suppress the growth of competitors (from Fig. 3). These experimental data resulted in a ranking, from least to most competitive, of *Porites* < *Cyphastrea* < *Acropora* < *Stylophora* < *Galaxea* < *Pocillopora* under ambient conditions and *Porites* < *Cyphastrea* < *Stylophora* < *Galaxea* < *Acropora* < *Pocillopora* under OA conditions (Table 3). An alternative way to determine the competitive hierarchy is based on the capacity of species to themselves avoid growth suppression in the presence of competitors (i.e., data presented in Fig. 4). In this case, we determined the competitive ranking by scoring each species pair based on the homogeneous subsets depicted in Fig. 4 (see Materials and Methods) which resulted in a ranking, from least to most competitive, of *Porites* < *Cyphastrea* < *Stylophora* < *Acropora* < *Galaxea* < *Pocillopora*. Across the five different competitive hierarchies, *Porites* and *Cyphastrea* were the least competitive in all scenarios (Table 3). *Stylophora* and *Galaxea* were intermediate in most scenarios, although their ranking shifted under OA compared with present-day conditions and *Galaxea* had the highest capacity to maintain growth in the presence of competitors. *Acropora* and *Pocillopora* were the highest ranked species, with *Acropora* having increased capacity to suppress the growth of its competitors under OA compared with ambient conditions (Table 3).

## Discussion

The results of this study indicate that increasing OA in the future may alter spatial competition dynamics between reef corals. Consistent with other reports, our results show differential susceptibility of the study species to OA conditions (significant species by treatment interactions, see Results), with *Porites, Pocillopora* and *Cyphastrea* the most tolerant, *Galaxea* the least tolerant, and *Stylophora* and *Acropora* intermediate between these taxa. These differences between species generally confirm the results of previous studies. For example, arborescent *Acropora* was more susceptible to OA than massive *Porites* and *Pocillopora* in one experimental study^41^, and *Galaxea* was highly susceptible to OA in another study^42^. These decreases in growth meant that the spatial competition models predicted a decrease in percentage cover of corals on reefs under OA compared with ambient conditions. In addition to decreased coral cover, slower growth rates can potentially lead to reduced life-time reproductive output (i.e., fitness) of colonies, because coral fecundity increases with colony size (e.g., ref. 37). Therefore, a likely decline in numbers of coral larvae transported within and between reefs is an indirect effect of reduced growth that was not incorporated into our model.

Despite decades of study of spatial competition on reefs, there are surprisingly few empirical studies that systematically quantify the relative effects of both intra- and interspecific competition. For instance, Rinkevich & Loya (1985) demonstrated that intraspecific competition suppresses growth of *S. pistillata* (consistent with our study), but did not quantify interspecific competition^43^. Conversely, Tanner (1997) showed that growth of *Acropora hyacinthus* and *P. damicornis* was suppressed under interspecific competition (again, consistent with our study), but did not quantify effects of intraspecific competition^44^. The only other studies, as far as we are aware, that have compared the effects of intra- and interspecific competition found comparable growth suppression under both types of competition instead of the much greater effect of interspecific competition observed here^31^,^32^,^45^. We suggest these contrasting results are due to differences in study duration; our competition trials ran for 1 year compared with 3–4 weeks in Evensen *et al*.^31^ and Evensen & Edmunds (2016)^32^. Comparison with these two studies indicates that, over a shorter time period, and before the tissues from competing colonies come into contact with each other, the effects of heterospecific and conspecific competitors are indistinguishable. Differences between studies might also be due to the broad taxonomic range of species used in our study compared to trials between two species from the same genus in Idjadi & Karlson (2007)^45^. Given that direct digestion of tissues of competing colonies is a common mechanism of coral competition^12^, it is possible that the ‘recognition’ of a competing colony as a heterospecific is less pronounced between closely related species.

For five of six study species, lowered pH lead to >25% reduction in growth rates in the absence of competition, but growth rates were similarly suppressed under ambient and OA conditions when in the presence of interspecific competition. Our results show no further significant growth reduction of interspecific competitors under acidified conditions, indicating that the OA effects are negligible when growth is already suppressed by competition. Growth under intraspecific competition was slower than in the absence of competition but, in contrast to interspecific competition, OA further suppressed growth of colonies competing with conspecifics by between 31 and 66% for five of six study species (all except *Porites*). When combined, these effects meant that projected coral cover based on a general spatial competition model was lower under OA compared with present-day conditions. The magnitude of the effect of each heterospecific competitor on each species was the same under OA and ambient conditions. Particular competitors (*Acropora, Pocillopora* and *Cyphastrea*), however, had a larger effect on the growth of conspecifics in acidified seawater. This lead to adjustments in the position of some species in the competitive hierarchy under the lowered pH conditions expected in the future.

Results of our study predict changes in the position of corals in the competitive hierarchy under pH conditions anticipated in the future; four of six coral species were ranked differently under present-day compared with OA conditions using two different competitive dominance metrics. This is consistent with other evidence of changing environmental drivers shifting competitive balances to favor certain species or growth forms over others. For instance, Genin *et al*.^46^ observed changes in the competitive superiority of *G. fascicularis* in the Gulf of Aqaba (GoA) and the Red Sea as a result of changing hydrodynamic conditions on the reef. Likewise, the seaweed *Caulerpa taxifolia* outcompetes the seagrass *Posidonia oceanica* in the Mediterranean Sea under high nutrient conditions^47^. Similar competitive shifts also occur among terrestrial plants. For example, elevated temperatures may benefit the competitive advantage of C4 plants^48^, whereas C3 plants may derive a competitive benefit following N deposition^49^.

Previous studies of coral competition have noted inconsistencies between competitive ability and species’ local abundances (e.g., refs 12 and 16). Similarly, the competitive hierarchy determined here is only partially consistent with the relative abundances of the study species in the GoA (Table 1). On coral reefs, competition typically occurs only on a spatial scale of cm (e.g., ref. 12), and the rates at which certain species encounter and compete with each other can be influenced by species-specific microhabitat preferences (e.g., ref. 29). Clearly, various factors influence the local abundances of coral species including their life-history strategies, environmental conditions (e.g., ref. 50) and stochastic environmental fluctuations (e.g., ref. 51). Nevertheless, our results show that incorporating the effects of OA on coral growth, and the interaction between environmental conditions and the presence of competitors, is important for predicting the relative abundances of corals on reefs in the future.

Worldwide coral cover has been declining (~5% per decade in the Indo-Pacific), primarily due to coral bleaching^28^, but also storms, crown-of-thorns starfish outbreaks^52^ and coral diseases^53^. As a result, space limitation on degraded reefs is potentially less severe than it was in the past, although our study refers to reefs that have hitherto suffered lesser damage and have relatively high coral cover. There is a general view that the effects of OA on competitive interactions may be heightened if corals are additionally stressed from high temperatures, and, hence, our findings might underestimate the effects likely to be seen in the field. Nevertheless, the high latitude reefs of the GoA experience cooler SST and are less affected by thermal stress^54^. OA, therefore, and other local stressors, are likely the dominant factors influencing future coral communities in this area.

Direct competitive interactions, both intra- and interspecific, are frequently observed on reefs^55^. Previous studies that have investigated changes in coral growth in response to OA have potentially overlooked long-term effects on coral populations by growing experimental coral fragments in isolation from each other or in short-term experiments. Our results point to more extreme OA effects on coral growth under intraspecific competition with 5 out of 6 of the study species. The effects of OA, however, are overwhelmed or irrelevant in the presence of interspecific competition. These findings underscore the importance of accounting for competition-dependent changes in coral growth when scaling up experimental studies to explain ecosystem functioning in the field. Collectively, our experimental and modeling results demonstrate that the outcome of interactions between coral colonies on reefs, both within and between species, will change as pH declines. Such changes may lead to a shift in species composition and biodiversity of coral assemblages on reefs and potentially impair reef functionality (e.g., lower structural complexity) and, as a consequence, alter the quality and quantity of reef ecological goods and services.

## Materials and Methods

### Experimental design

The study was carried out in a seawater flow-through system at the Interuniversity Institute (IUI) for Marine Sciences in Eilat (GoA, Red Sea, 29°30′N, 34°55′E). Seawater pH was regulated using a pH controller (Aquastar, Germany), connected to pH electrodes located in 1000 L mixing tanks and calibrated using the National Bureau of Standards (NBS) scale. pH was manipulated by bubbling pure CO_2_ into the mixing tanks to attain the desired pH, and then supplied via tubing to the various aquaria for each pH treatment. Six common Indo-Pacific coral species were examined: *Galaxea fascicularis, Pocillopora damicornis, Acropora variabilis, Cyphastrea chalcidicum, Stylophora pistillata* and *Porites lutea*. Six colonies of each species were collected in June 2012 from the reef in front of the IUI at 10 m depth. All the colonies were fragmented to have equally sized corals (~2 cm diameter) and tagged to account for parent colony. Considering the long-term study was planned for the duration of one year, we chose to use relatively small coral pieces at the beginning of the experiment, allowing them to grow throughout the experimental period and subsequently engage in competitive interactions for space. The size of the corals at the experiment start also corresponds to relatively newly-settled coral recruits/juveniles in the coral reef which encounter either con- or heterospecific competitors. A total of 84 fragments per species was prepared (14 fragments per colony). In order to mimic the natural conditions of interaction as much as possible, corals were positioned on pre-labeled glass slides (with a designated central focal line) with a gap of 1 cm between the two opponents using super glue (Henkel Loctite Ltd.). A total of 15 pair-wise interactions were prepared with the six coral species, as well as intraspecific pairing and non-interacting single corals. Fragments were positioned such that growth over the following year would result in a competitive interaction as the corals grew laterally on the glass slide.

After a one-month recovery period, all pair-wise interactions and single fragments (without competition) were evenly divided in twelve 30 L tanks (six tanks per treatment; in each individual tank, fragments from the same parent colony were used for each interspecific/intraspecific interaction or single coral, with two identical tanks between pH treatments; each pair-wise interaction/single coral had six replicates per pH treatment) and supplied with running seawater (0.5 L min^−1^) at two different pH levels: (i) pH 8.1 (ambient; present-day) and (ii) pH 7.6 [reduced pH; representing upper-threshold for the IPCC “business-as-usual” scenario (RCP 8.5) for 2100^35^; the RCP 8.5 scenario in this study refers only to pH predictions and not added temperature changes]. Corals were maintained in their respective pH treatment under ambient seawater temperature (ranging 21.6–26.3 °C as measured in the aquaria during the experimental period; comparable to long-term SST for that time of the year in the GoA). Light (250 ± 20 μmol quanta m^−2 ^s^−1^, 12 L:12D photoperiod) was provided by two metal halide lamps (400 W/D, Osram GmBH, Germany). Submersible pumps ensured water mixing in the tanks. Corals were fed once a week with *Artemia salina* nauplii (400,000 per aquarium) for the entire duration of the experiment.

### Carbonate chemistry

Monitoring software (Aquastar, Germany) in the pH system showed that daily pH variability was low (±0.05 pH units) throughout the experiment. Temperature and pH_NBS_ in the aquaria were measured daily (CyberScan pH 11; Eutech Instruments Pte Ltd, Singapore). Total alkalinity (TA) in the aquaria and mixing tanks was measured regularly using a Metrohm 862 compact titrosampler^56^. *p*CO_2_, dissolved inorganic carbon, HC0_3_^−^, CO_3_^2−^, CO_2(aq)_ and Ω_arag_ were calculated from the pH_NBS_ and TA measurements using the program CO_2_SYS^57^, selecting the constants of 58. Experimental seawater parameters are shown in Table S1.

### Growth estimations

Our measurements do not describe the competitive mechanisms employed by each study species to compete for space, but interpret change in overall colony surface area as a measure for competitive ability, since it is the end-result of the competitive interaction for space between two corals, i.e., the ability to overgrow/overtop on another or be overgrown/overtopped, as well as other means, such as digestive aggression causing partial mortality of colonies and loss of space on the reef.

Photos were taken at various time intervals during the experiment by digital camera at a fixed distance using a scale (CoolPix 8400, Nikon, Japan) to measure coral growth, i.e., changes in overall colony surface dimensions. We used changes in total coral colony living tissue surface area as our metric of competition ability because it encapsulates both tissue growth and tissue loss through digestion, and because it enables comparison of the overall amount of new reef ‘framework’ generated by corals with different colony morphologies^33^. By this metric, a colony that is decreasing in size over time is losing tissue through digestion or partial mortality faster than it is able to produce new tissue. Other methods for measuring effects of competition on growth, such as measuring changes in ‘area of occupancy’, measure the horizontal area of space occupied by a colony (e.g., refs 33 and 59), but do not indicate vertical growth, and this can bias measurement of the effects of competition on growth for different coral colony morphologies. Similarly, measuring effects of competition by measuring overgrowth along the margin of contact between competing corals can misrepresent the effects of competition because corals grow in multiple directions. Finally, for corals in the natural environment, fecundity is directly associated with tissue surface area (number of polyps) and, therefore, measuring effects of competition on coral surface area provides a clearer indication of changes in fitness compared with these other metrics. For the purposes of this study, we do not differentiate between tissue and skeleton extension, although we recognize that both are functionally and mechanistically intertwined.

Three sets of photographs were taken: the first covered 360° of the corals while the camera was parallel to the glass slide. The second set of photographs was at multiple angles with the camera at *c*. 45° angle to the glass slide. The third set of photographs repeated the circular photography as above, but with the camera at *c*. 90° angle to the glass slide. Depending on coral morphology, 5–15 images were taken in the circular shooting, with more photographs for the more complex forms such as the branching *Pocillopora, Stylophora* and *Acropora*. All images were then analyzed with CPCe 4.0 (NCRI, USA) image analysis software^60^ to measure dimensional parameters of the corals (length, height, radius and diameter).

Net coral growth (surface area growth of new tissue minus loss of tissue due to competitor presence; competitive mechanisms observed during the experiment included planar overgrowth of one species by the other or partial mortality from digestive aggression) was measured from day 1 to day 345 for all the species included in the pair-wise interactions (*n* = 6 per interaction/per pH treatment). Growth estimations of single coral fragments (without competition) were also measured (*n* = 6 per species/per pH treatment). Surface area estimations of the corals were performed using geometric measurements given the non-invasive and highly accurate nature of this technique^61^. Each coral was divided into several sections and assigned an approximate geometric form or shape to each, after which single measurements of dimensional parameters for each section were calculated using their respective surface area equations. The surface area of the entirety of branches of *Acropora, Stylophora* and *Pocillopora* colonies was calculated as cylinder shell surfaces. The radius and height of branches were assessed by measuring the branch diameter at the base of each branch and the height from branch base to tip. Calculated surface areas from all branches were added to gain the total coral surface area estimate. Colonies of the massive corals *Galaxea, Cyphastrea* and *Porites* were interpreted as hemispheres. Maximum and minimum horizontal diameters of each colony were measured, and the average radius was calculated. The height of the colony was assessed from the glass slide plane to the highest point of the coral colony. Thereafter, colony surface area was calculated by the use of the surface area formula for hemispheres.

Use of digital image analysis was verified by physical measurements, which differed by less than 5%. In addition, the maximum diameter of branching colonies (*Acropora, Stylophora* and *Pocillopora*) was used for estimation of horizontal area occupied/overtopped in modeling analyses.

### Statistical analyses

Statistical analyses were conducted in R^62^. A three-way ANOVA, including all interaction terms, was used to test whether and how different types of competition (intraspecific versus interspecific, categorical fixed factor) affected the growth of different species (categorical fixed factor) under different pH treatments (ambient versus future, categorical fixed factor). Subsequently, for the colonies involved in interspecific competition, three-way ANOVA, including all interaction terms, was used to test whether and how different heterospecific competitors (categorical fixed factor) affected the growth of different species (categorical fixed factor) under different pH treatments (ambient versus future, categorical fixed factor). In both of these analyses, ‘Tank’ was initially included as a random effect (using a linear mixed effects model) but was subsequently removed because analyses including tank did not explain significantly more of the variance in our data (Likelihood ratio tests; 1) comparing ANOVA for ‘types of competition’ with same model including tank as a random effect; likelihood ratio = 1.46, p = 0.23; 2) comparing ANOVA for ‘identity of competitor’ with same model including tank as a random effect; likelihood ratio = 0.001, p = 0.99). All data analyses were checked for normality by visual inspection of residuals and data were log-transformed when required. Post-hoc Tukey’s tests, which account for family-wise error due to multiple comparisons, were performed to identify the groups that were significantly different from each other when significant effects were detected. Throughout the paper, results were considered significant for a *p* value < 0.05 and, unless otherwise specified, mean values are presented ± SEM.

### Model description

The model of general spatial competition consists of a set of coupled differential equations (Equations 1 and 2), one per species, that describes the proportion of space occupied by each species in a homogenous environment that is assumed to have constant environmental conditions through time (see ref. 39). The model incorporates competition between species via overgrowth, which is a type of interference competition where the amount of space one species loses is equal to the amount of space gained by the other species. In our specific case, we consider overgrowth competition to occur both via direct overgrowth of tissues and by overtopping. As the model specifically considers competition, and does not allow for facilitation, 0 ≥ *c*_*ij*_ ≥ 1. The overgrowth competitiveness depends on the balance of growth of species *i* along the boundary with *j* and the growth of species *j* along the boundary with *i*, values that can be expected to differ as they depend upon species-specific growth rates. Hence, the net expansion of competing species is expressed as *c*_*ij*_*b*_*i*_ − *c*_*ji*_*b*_*j*_. The coupled equations are then expressed as:


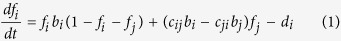



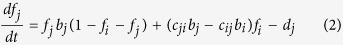


where *f* is the fraction of space occupied by each species in the environment at any point in time.

Each of the model parameters were estimated for each species using the experimental data and we analyzed the parameterized model to reveal the space occupancy of each species, *f*_*i*_ and *f*_*j*_, present in the 2-species community through time. Species-specific expansion rate, *b*_*i*_, was estimated from measurements of the growth of colonies in the absence of competitors. Species-specific mortality rate, *d*_*i*_, was conservatively set as constant for all species and was chosen to be lower than the minimum observed growth rate to ensure that all species could, in principle, grow and expand in the modeled environment. The overgrowth coefficients, *c*_*ij*_ and *c*_*ji*_, were estimated separately for each species in competition with all other heterospecifc competitors, and parameter estimates were calculated from proportional growth of colonies under competition relative to growth in the absence of competition. For both *c* and *b*, values, which were originally measured as change in tissue surface area, were converted to horizontal projected area of the colony based on colony geometry. This metric captures the expansion of colonies over the horizontal area of the habitat rather than the change in tissue area. The conversion was made by assuming a hemispherical colony morphology for *Cyphastrea, Galaxea* and *Porites* whereby horizontal area, h = πr^2^ where r, radius, is calculated from the colony surface area as r = (surface area/2π)^0.5^. For *Acropora, Pocillopora* and *Stylophora*, horizontal projected area was calculated as previously except that radius was estimated directly from the images of the colonies, and was measured at the maximum radius of each colony so as to capture the projected area of the colony rather than the area of the branch base.

## Additional Information

**How to cite this article**: Horwitz, R. *et al*. Spatial competition dynamics between reef corals under ocean acidification. *Sci. Rep.* **7**, 40288; doi: 10.1038/srep40288 (2017).

**Publisher's note:** Springer Nature remains neutral with regard to jurisdictional claims in published maps and institutional affiliations.

## Supplementary Material

Supplementary Information

## Figures and Tables

**Table 1:** Description of the study species.

Species	Colony morphology	Relative abundance (% cover)	Aggression rank
*Acropora variabilis*	Branching	18	3
*Stylophora pistillata*	Branching	11	2
*Cyphastrea chalcidicum*	Mound-shaped	4	4
*Porites lutea*	Mound-shaped	4	1
*Pocillopora damicornis*	Branching	2	5
*Galaxea fascicularis*	Encrusting to mound-shaped	1	6

The aggression ranking of species (1 = least aggressive, 6 = most aggressive) is modified from Abelson and Loya (1999)^16^. Relative abundance data are from survey data collected by the National Monitoring Program in the Gulf of Aqaba (NMP available from http://www.iui-eilat.ac.il/NMP/).

**Table 2:** Variance analysis of effects of competition and pH treatment on coral growth.

Effect	Df	F	p
*Type of competition* (*no competitor compared with intra- and interspecific competition*)			
pH Treatment	1, 180	895	<0.001
Species	5, 180	1124	<0.001
Type of competition	2, 180	3402	<0.001
Treatment x Species	5, 180	14	<0.001
Treatment x Competition	2, 180	91	<0.001
Species x Competition	10, 180	63	<0.001
Treatment x Species x Competition	10, 180	8.3	<0.001
*Identity of competitor*			
pH Treatment	1, 300	56	<0.001
Species	5, 300	256	<0.001
Competitor	5, 300	196	<0.001
Treatment x Species	5, 300	3.9	<0.01
Treatment x Competitor	5, 300	4.0	<0.01
Species x Competitor	19, 300	15	<0.001
Treatment x Species x Competitor	19, 300	1.1	0.39

In ‘Type of competition’ data are averaged across the different heterospecific competitors for each species in each of six replicate aquaria per pH treatment.

**Table 3:** Competitive hierarchy of study species under different conditions (lowered pH ‘OA’ and present-day pH ‘Ambient’) based on different scenarios.

Species	Spatial competition model		Capacity to suppress growth of competitors		Capacity to resist competitors
	Ambient	OA	Ambient	OA	—
*Pocillopora damicornis*	6	**5**	6	6	5
*Acropora variabilis*	5	**6**	3	5	4
*Galaxea fascicularis*	4	**3**	5	**4**	6
*Stylophora pistillata*	3	**4**	4	**3**	3
*Cyphastrea chalcidicum*	2	2	2	2	2
*Porites lutea*	1	1	1	1	1

Bold values highlight shifts in the competitive hierarchy under OA versus Ambient conditions.
