# Supplementary material for: Spatial competition dynamics between reef corals under ocean acidification
Source: Sci Rep. 2017 Jan 9;7:40288. doi: 10.1038/srep40288 (PMC5220319; doi:10.1038/srep40288)
Supplement: Supplementary Information [file srep40288-s1.pdf]

Spatial competition dynamics between reef corals under ocean acidification

Rael Horwitz*[1,2,γ], Mia O. Hoogenboom[3, γ], Maoz Fine[1,2]

[1]*The Mina and Everard Goodman Faculty of Life Sciences, Bar-Ilan University, Ramat-Gan 52900, Israel*

[2]*The Interuniversity Institute for Marine Sciences, Eilat 88103, Israel*

[3] *College of Science and Engineering and ARC Centre of Excellence for Coral Reef Studies, James Cook University, Townsville, Queensland 4811, Australia*

γ The first two authors made an equal contribution to writing this paper.

*Corresponding author:

Rael Horwitz

Telephone: +689 (89) 598599

Fax: +689 (40) 56 28 15

Email: horwitzrael@gmail.com

SI1: Matlab code and parameter estimates for spatial competition model.

```
% Matlab Script
% Mia Hoogenboom
% 10 August 2015

% Objective = run 2 species general spatial competition model for corals
% based on data from Rael Horwitz and Maoz Fine from experimental work in the Gulf of Aqaba

% Corals were grown under control or high pCO2 (low pH) treatment in pairwise
% competition interactions under both intraspecifc and interspecific
% competition. Corals were also grown individually (no competition)
% Experiments were conducted on 6 species
        % Acropora variabilis
        % Cyphastrea chalcidicum
        % Galaxea fascicularis
        % Pocillopora damicornis
        % Porites lutea
        % Stylophora pistillata

% Mathematical model is as described by:
% Crowley PH, Davis HM, Ensminger AL, Fuselier LC, Jackson JK, McLetchie DN (2005)
% “A general model of local competition for space” Ecology Letters 8:176-188

%____________________________________________________________
% COMPARE LONG-RUN POPULATION SIZES FOR SPECIES INVOLVED
% IN PAIRWISE COMPETITIVE INTERACTIONS USING A 2-SPECIES
% SYSTEM OF COUPLED DIFFERENTIAL EQUATIONS

[t,n]=ode45('gencomp2',[0,50],[0.4,0.4]);
% Runs numerical evaluation of the coupled differential equations
% initialising with population sizes (% cover) of both species = 40%
% runs for 50 model timesteps = years given the data
% Where ‘gencomp2’ is a function as below

plot(t,n)
% plots the model outputs

ylim([0,1])
% sets the y-axis limits between 0 - 1

legend('Sp 1','Sp 2')
% plots the legend on the figure
n
% outputs long-run proportional cover for the two species
```

```
function ftime=gencomp2(t,f)
% coupled differential equations for general spatial competition
% 2 species, pairwise comparisions

b = [7.49,11.99];
% per capita expansion rate onto unoccupied space (b > 0)
% ordered alphabetically Acropora, Cyphastrea, Galaxea, Pocillopora, Porites, Stylophora
% values are measured as the proportional change in surface area in the
% absence of competition, values adjusted from surface area to horizontal planar area based on
% colony shape
% b1 = [14.97   4.21    7.42    7.49    1.68    11.99]; CONTROL CONDITIONS
% b2 = [10.97   3.03    2.89    5.32    1.25    8.45]; HIGH pCO2

cmat = [0,0.08;0.25,0];
% dimensionless overgrowth coefficients (0 < c < 1)
% values are ordered alphabetically in rows and columns, values indicate the decrease in growth %
in the presence of competitors relative to growth in the absence of competitors
% values on the main diagonal are set to zero
% cmat1 = [0        0.32    0.24    0.26    0.15    0.12;
%           0.19    0       0.32    0.28    0.26    0.20;
%           0.07    0.08    0       0.19    0.06    0.08;
%           0.06    0.07    0.14    0       0.09    0.01;
%           0.21    0.60    0.34    0.34    0       0.24;
%           0.18    0.43    0.25    0.30    0.20    0]; CONTROL CONDITIONS
%
% cmat2 = [0        0.22    0.12    0.20    0.12    0.14;
%           0.22    0       0.28    0.29    0.24    0.23;
%           0.09    0.05    0       0.33    0.02    0.11;
%           0.07    0.04    0.00    0       0.05    0.01;
%           0.29    0.62    0.36    0.35    0       0.29;
%           0.17    0.43    0.23    0.29    0.15    0]; HIGH PCO2 CONDITIONS

d = [1,1];
% per capita mortality rate (d >= 0)
% ordered alphabetically Acropora, Cyphastrea, Galaxea, Pocillopora, Porites, Stylophora
% assuming that background mortality is equivalent for all species
% value set to be lower than the smallest value of 'b' so that births >
% deaths and species can, in principle, expand in the environment


ftime(1,1) = f(1) * ((b(1)*(1 - f(1) - f(2))) + (cmat(2,1)*b(1) - cmat(1,2)*b(2))*f(2) - d(1));
ftime(2,1) = f(2) * ((b(2)*(1 - f(1) - f(2))) + (cmat(1,2)*b(2) - cmat(2,1)*b(1))*f(1) - d(2));

end
```

Table S1 Carbonate chemistry of seawater. Carbonate chemistry in present-day (pH 8.1) and acidified (pH 7.6) treatments was calculated from pH_{NBS}, total alkalinity (TA), ambient seawater temperature and salinity (40 ppm) using the program CO_2SYS[57]. All data shown are the mean (± SD). Dissolved inorganic carbon (DIC); aragonite saturation state (Ω_{arag}).

Treatment	pH_{NBS}	TA (µeq kg^{-1})	pCO_2 (µatm)	DIC (µmol kg^{-1})	$HC0_3^-$ (µmol kg^{-1})	CO_3^{2-} (µmol kg^{-1})	$CO_{2(aq)}$ (µmol kg^{-1})	Ω_{arag}
Ambient pH	8.18 (0.01)	2501.72 (1.2)	400.27 (2.46)	2145.7 (11.07)	1883.19 (18.59)	251.13 (7.87)	11.37 (0.33)	4.02 (0.03)
Decreased pH	7.62 (0.02)	2504.91 (3.63)	1795.39 (7.4)	2434.69 (6.8)	2300.08 (8.84)	83.58 (3.98)	51.02 (1.67)	1.26 (0.06)

Table S2 Multiple pair-wise comparisons between treatment groups based on Tukey's HSD for each species and each treatment combination, where 'difference' is the mean difference in growth of each species between each pair of competitors, lower and upper bounds give the 95% confidence interval of the difference and probability that the difference is greater than zero (p).

**Species**	**Treatment**	**Other treatment comparison**	**Difference**	**Lower bound**	**Upper bound**	**p**
Acropora	Interspecific	OA - Control	-0.18	-0.52	0.17	0.99
Acropora	None	OA - Control	-1.00	-1.34	-0.65	<0.001
Acropora	Intraspecific	OA – Control	-1.27	-1.62	-0.93	<0.001
Acropora	Control	None - Intraspecific	1.37	1.02	1.71	<0.001
Acropora	Control	Intraspecific - Interspecific	1.64	1.29	1.98	<0.001
Acropora	Control	None - Interspecific	3.00	2.66	3.35	<0.001
Acropora	OA	Intraspecific - Interspecific	0.54	0.20	0.88	<0.001
Acropora	OA	None - Interspecific	2.18	1.84	2.52	<0.001
Acropora	OA	None - Intraspecific	1.64	1.30	1.99	<0.001
Cyphastrea	Interspecific	OA - Control	-0.41	-0.75	-0.06	<0.001
Cyphastrea	None	OA - Control	-0.66	-1.00	-0.31	<0.001
Cyphastrea	Intraspecific	OA – Control	-0.55	-0.89	-0.20	<0.001
Cyphastrea	Control	Intraspecific - Interspecific	0.95	0.61	1.30	<0.001
Cyphastrea	Control	None - Interspecific	1.95	1.61	2.30	<0.001
Cyphastrea	Control	None - Intraspecific	1.00	0.65	1.34	<0.001
Cyphastrea	OA	None - Intraspecific	0.88	0.54	1.23	<0.001
Cyphastrea	OA	Intraspecific - Interspecific	0.82	0.47	1.16	<0.001
Cyphastrea	OA	None - Interspecific	1.70	1.36	2.04	<0.001
Galaxea	Intraspecific	OA - Control	-0.89	-1.24	-0.55	<0.001
Galaxea	None	OA - Control	-0.99	-1.33	-0.64	<0.001
Galaxea	Interspecific	OA - Control	-0.35	-0.69	0.00	0.05
Galaxea	Control	Intraspecific - Interspecific	0.80	0.45	1.14	<0.001
Galaxea	Control	None - Intraspecific	0.81	0.47	1.15	<0.001
Galaxea	Control	None - Interspecific	1.61	1.26	1.95	<0.001
Galaxea	OA	Intraspecific - Interspecific	0.25	-0.10	0.59	0.63
Galaxea	OA	None - Interspecific	0.97	0.62	1.31	<0.001
Galaxea	OA	None - Intraspecific	0.72	0.37	1.06	<0.001
Pocillopora	Intraspecific	OA - Control	-1.01	-1.35	-0.66	<0.001
Pocillopora	None	OA - Control	-0.70	-1.05	-0.36	<0.001
Pocillopora	Interspecific	OA - Control	-0.13	-0.47	0.22	1.00
Pocillopora	Control	Intraspecific - Interspecific	1.10	0.76	1.44	<0.001
Pocillopora	Control	None - Intraspecific	1.37	1.02	1.71	<0.001

Pocillopora	Control	None - Interspecific	2.47	2.12	2.81	<0.001
Pocillopora	OA	Intraspecific - Interspecific	0.22	-0.12	0.56	0.84
Pocillopora	OA	None - Interspecific	1.89	1.55	2.24	<0.001
Pocillopora	OA	None - Intraspecific	1.67	1.33	2.02	<0.001
Porites	Intraspecific	OA - Control	-0.24	-0.58	0.10	0.69
Porites	None	OA - Control	-0.37	-0.71	-0.03	0.02
Porites	Interspecific	OA - Control	-0.26	-0.61	0.08	0.47
Porites	Control	Intraspecific - Interspecific	0.83	0.49	1.17	<0.001
Porites	Control	None - Intraspecific	0.79	0.45	1.13	<0.001
Porites	Control	None - Interspecific	1.62	1.28	1.97	<0.001
Porites	OA	Intraspecific - Interspecific	0.85	0.51	1.20	<0.001
Porites	OA	None – Intraspecific	0.66	0.32	1.01	<0.001
Porites	OA	None - Interspecific	1.51	1.17	1.86	<0.001
Stylophora	None	OA - Control	-1.01	-1.35	-0.66	<0.001
Stylophora	Interspecific	OA - Control	-0.04	-0.38	0.31	1.00
Stylophora	Intraspecific	OA - Control	-1.14	-1.48	-0.79	<0.001
Stylophora	Control	None - Intraspecific	1.72	1.37	2.06	<0.001
Stylophora	Control	Intraspecific - Interspecific	1.85	1.50	2.19	<0.001
Stylophora	Control	None - Interspecific	3.57	3.22	3.91	<0.001
Stylophora	OA	None - Intraspecific	1.85	1.51	2.19	<0.001
Stylophora	OA	Intraspecific - Interspecific	0.75	0.40	1.09	<0.001
Stylophora	OA	None - Interspecific	2.60	2.25	2.94	<0.001

Table S3 Multiple pair-wise comparisons between treatment groups based on Tukey's HSD for each species and each pair of competitors. Pair-wise comparisons show the effects of one competitor on each species relative to the effects of another competitor where 'difference' is the mean difference in growth of each species between each pair of competitors, lower and upper bounds give the 95% confidence interval of the difference and probability that the difference is greater than zero (p).

**Species**	**Effect of competitor A on Species compared with effect of competitor B on Species**	**Difference**	**Lower bound**	**Upper bound**	**p**
Acropora	Acropora:Galaxea-Acropora:Cyphastrea	-0.82	-1.23	-0.42	<0.001
Acropora	Acropora:Pocillopora-Acropora:Cyphastrea	-0.90	-1.30	-0.50	<0.001
Acropora	Acropora:Pocillopora-Acropora:Galaxea	-0.08	-0.48	0.33	1.00
Acropora	Acropora:Porites-Acropora:Cyphastrea	0.13	-0.28	0.53	1.00
Acropora	Acropora:Porites-Acropora:Galaxea	0.95	0.55	1.35	<0.001
Acropora	Acropora:Porites-Acropora:Pocillopora	1.03	0.62	1.43	<0.001
Acropora	Acropora:Stylophora-Acropora:Cyphastrea	-0.33	-0.74	0.07	0.34
Acropora	Acropora:Stylophora-Acropora:Galaxea	0.49	0.09	0.90	<0.001
Acropora	Acropora:Stylophora-Acropora:Pocillopora	0.57	0.16	0.97	<0.001
Acropora	Acropora:Stylophora-Acropora:Porites	-0.46	-0.86	-0.05	0.01
Cyphastrea	Cyphastrea:Galaxea-Cyphastrea:Acropora	-0.98	-1.39	-0.58	<0.001
Cyphastrea	Cyphastrea:Pocillopora-Cyphastrea:Acropora	-1.07	-1.48	-0.67	<0.001
Cyphastrea	Cyphastrea:Pocillopora-Cyphastrea:Galaxea	-0.09	-0.49	0.32	1.00
Cyphastrea	Cyphastrea:Porites-Cyphastrea:Acropora	0.69	0.28	1.09	<0.001
Cyphastrea	Cyphastrea:Porites-Cyphastrea:Galaxea	1.67	1.27	2.08	<0.001
Cyphastrea	Cyphastrea:Porites-Cyphastrea:Pocillopora	1.76	1.35	2.16	<0.001
Cyphastrea	Cyphastrea:Stylophora-Cyphastrea:Acropora	0.41	0.01	0.82	0.04
Cyphastrea	Cyphastrea:Stylophora-Cyphastrea:Galaxea	1.40	0.99	1.80	<0.001
Cyphastrea	Cyphastrea:Stylophora-Cyphastrea:Pocillopora	1.49	1.08	1.89	<0.001
Cyphastrea	Cyphastrea:Stylophora-Cyphastrea:Porites	-0.27	-0.68	0.13	0.78
Galaxea	Galaxea:Stylophora-Galaxea:Acropora	0.15	-0.25	0.56	1.00
Galaxea	Galaxea:Stylophora-Galaxea:Cyphastrea	-0.23	-0.63	0.18	0.97
Galaxea	Galaxea:Stylophora-Galaxea:Pocillopora	0.52	0.12	0.93	<0.001
Galaxea	Galaxea:Stylophora-Galaxea:Porites	-0.28	-0.68	0.13	0.74
Galaxea	Galaxea:Porites-Galaxea:Acropora	0.43	0.03	0.84	0.02
Galaxea	Galaxea:Porites-Galaxea:Cyphastrea	0.05	-0.35	0.46	1.00
Galaxea	Galaxea:Porites-Galaxea:Pocillopora	0.80	0.40	1.21	<0.001

Galaxea	Galaxea:Pocillopora-Galaxea:Acropora	-0.37	-0.78	0.03	0.13
Galaxea	Galaxea:Pocillopora-Galaxea:Cyphastrea	-0.75	-1.16	-0.35	<0.001
Galaxea	Galaxea:Cyphastrea-Galaxea:Acropora	0.38	-0.02	0.78	0.11
Pocillopora	Pocillopora:Stylophora-Pocillopora:Acropora	0.18	-0.22	0.59	1.00
Pocillopora	Pocillopora:Stylophora-Pocillopora:Cyphastrea	0.02	-0.38	0.43	1.00
Pocillopora	Pocillopora:Stylophora-Pocillopora:Galaxea	0.18	-0.22	0.59	1.00
Pocillopora	Pocillopora:Stylophora-Pocillopora:Porites	-0.14	-0.54	0.27	1.00
Pocillopora	Pocillopora:Galaxea-Pocillopora:Acropora	0.00	-0.41	0.40	1.00
Pocillopora	Pocillopora:Galaxea-Pocillopora:Cyphastrea	-0.16	-0.56	0.25	1.00
Pocillopora	Pocillopora:Porites-Pocillopora:Cyphastrea	0.16	-0.24	0.57	1.00
Pocillopora	Pocillopora:Porites-Pocillopora:Galaxea	0.32	-0.08	0.72	0.42
Pocillopora	Pocillopora:Porites-Pocillopora:Acropora	0.32	-0.09	0.72	0.43
Pocillopora	Pocillopora:Cyphastrea-Pocillopora:Acropora	0.16	-0.25	0.56	1.00
Porites	Porites:Pocillopora-Porites:Acropora	-0.28	-0.69	0.12	0.70
Porites	Porites:Pocillopora-Porites:Cyphastrea	-0.61	-1.01	-0.20	<0.001
Porites	Porites:Pocillopora-Porites:Galaxea	0.14	-0.27	0.54	1.00
Porites	Porites:Stylophora-Porites:Acropora	0.12	-0.29	0.52	1.00
Porites	Porites:Stylophora-Porites:Cyphastrea	-0.21	-0.61	0.20	0.99
Porites	Porites:Stylophora-Porites:Galaxea	0.54	0.13	0.94	<0.001
Porites	Porites:Stylophora-Porites:Pocillopora	0.40	0.00	0.81	0.05
Porites	Porites:Galaxea-Porites:Acropora	-0.42	-0.82	-0.02	0.03
Porites	Porites:Galaxea-Porites:Cyphastrea	-0.75	-1.15	-0.34	<0.001
Porites	Porites:Cyphastrea-Porites:Acropora	0.33	-0.08	0.73	0.38
Stylophora	Stylophora:Porites-Stylophora:Acropora	0.77	0.37	1.17	<0.001
Stylophora	Stylophora:Porites-Stylophora:Cyphastrea	0.33	-0.08	0.73	0.38
Stylophora	Stylophora:Porites-Stylophora:Galaxea	0.96	0.56	1.37	<0.001
Stylophora	Stylophora:Porites-Stylophora:Pocillopora	2.10	1.70	2.50	<0.001
Stylophora	Stylophora:Pocillopora-Stylophora:Acropora	-1.33	-1.73	-0.93	<0.001
Stylophora	Stylophora:Pocillopora-Stylophora:Cyphastrea	-1.77	-2.18	-1.37	<0.001
Stylophora	Stylophora:Pocillopora-Stylophora:Galaxea	-1.14	-1.54	-0.73	<0.001
Stylophora	Stylophora:Galaxea-Stylophora:Acropora	-0.19	-0.60	0.21	1.00
Stylophora	Stylophora:Galaxea-Stylophora:Cyphastrea	-0.64	-1.04	-0.23	<0.001
Stylophora	Stylophora:Cyphastrea-Stylophora:Acropora	0.44	0.04	0.85	0.01
